# Everybody's Page

**Published:** 1892-04-16

**Authors:** 


### Everybody's Page.

Phosphorus poisoning, which is by no means uncommon, can, it is asserted, be combated successfully with a solution of two to five parts of permanganate of potash to a thousand of water. The remedy, which is said to be well borne by the stomach, has the additional advantage of being simple.

No one has ever doubted that to read and digest Shakespeare is a liberal education in itself, but the Theatres and Music Halls Committee were scarcely prepared to find that the immortal Bard's services may yet be pressed into the use of those who attempt to cure drunkenness. That his services would be of some value in this direction is evident from Mr. Irving's statement that the "Bells," when acted, produced a great consumption of brandy, while Shakespeare is nourished chiefly on soda-water.

The idea which has been put forward that an increase in the longevity of clergymen is due to the spread of the temperance movement amongst them is rather a slur upon those of a past generation. The clergy of the present day have undoubtedly taken a strong position in the temperance question, but as apostles rather than disciples; and, if there is a diminished mortality now amongst the clergy compared with a generation ago, it seems much more reasonable to suppose that they, like other people, are reaping the benefit of improved sanitation.

An interesting discussion took place at a recent meeting of the New York Academy of Medicine on the question of hypertrophy of the tonsils, the general view taken of the question being in favour of abolishing this cumbersome and too often irritating excrescence. The existence of a tonsil should, as one physician held, be regarded as a disease to be dealt with summarily and promptly, though he does not think that snoring is to be attributed to enlarged tonsils so much as to enlarged glands in the pharynx. Be this as it may, we are all anxious to deal summarily and promptly with a snore, though our efforts are too often unavailing.

A correspondent in the *New York Medical Record* sings the praises of the Trans-Pecos country in North-Western Texas as a health resort for consumptives. The chief claim of this district seems to be its dryness, which fully counterbalances the changes from heat to cold, and vice versâ. The humidity which exists in more equable climates is not, now at any rate, considered beneficial for invalids of this class, and the Trans-Pecos country has one characteristic which will recommend it strongly to English invalids, the existence of not more than two or three cloudy days in a month. With us, alas! the very reverse holds good almost literally.

The question of "hours of labour" does not seem likely to find a satisfactory solution just at present. Those who are most interested in the matter are divided in their opinion as to the advisability of shortening hours of labour on the one hand, and of accomplishing this end, if held desirable, by means of legislation on the other. Those who have given thought to the subject seem fairly unanimous in holding that eight hours of work per day for women should not be exceeded, and that labour beyond that limit is injurious to them. When it is remembered that the work of so many women is done under the most adverse circumstances as regards air and space, there can be little doubt of the correctness of this view. But, unfortunately, any attempt at reducing the working hours of women seems likely to meet with opposition on the ground that such a measure will cheapen their labour.

Notwithstanding all that has been done for Nice in the way of drainage by its enterprising Municipality, the question of sewage disposal seems to be a problem still to be satisfactorily solved. The method of house-drainage is alleged to be of the most antiquated order, and leaseholders have been as supine as the Municipality has been energetic. A good main drainage is practically useless if householders neglect the connecting drains.

With what most people will consider laudable zeal, the London School Board are most anxious to inculcate habits of thrift and temperance in the minds of the young, though opinions may differ as to the mode of operation which they recommend. It is not easy to make books on temperance as attractive as ordinary books of adventures and perils, and there is the danger, not very remote, of books which are intended to instil a wholesome train of thought having the opposite effect. If the child once looks upon the book as a "medicine" rather than a pleasure, his ideas on thrift, &c., may, from association, take a wrong turn.

The much-vexed question of the proprietorship of prescriptions, to which the medical practitioner, the patient, and dispensing chemist each lay claim, is decided by some people in favour of the practitioner on the simple ground that the prescription is the doctor's written instruction to the chemist. The opinion that the fact that prescriptions are written in Latin is conclusive evidence that they are not for the patient, because the average patient does not understand Latin, or what passes for Latin, is not worth serious consideration. It would be as reasonable and as logical to hold that a painting does not belong to its purchaser because the average purchaser of paintings does not know how to handle a brush.

Two gentlemen have come forward with a novel and convincing reason—convincing anyhow to themselves—for the substitution of vegetarian for meat diet. One suffered from chronic toothache, which has disappeared completely under the soothing and magic influence of a four years' vegetarian regimen; another has found the diet equally efficacious in its effect upon bronchitis. Some people may doubt whether there is the close connection between cause and effect assumed by these enthusiastic apostles of vegetarianism. Unfortunately we do not know whether Nebuchadnezzar enjoyed unusually good health when under compulsory abstinence from flesh, or valuable light would doubtless be thrown on the question.

They say that in England an old custom, however foolish, dies hard. We think one more foolish perhaps than any is dying now—the excess to which burial and mourning are carried. This custom had for its origin a wish to pay respect to loved ones gone, but its final development has become a meaningless parade—a useless waste of much-needed money. Private nurses have seen plenty of this, and see too much of it still—the effort it is often to put a whole family into voluminous black clothing, and provide a "suitable" cortège for the body of one who would have wished to be put to rest as simply as is possible. We can all of us do a little to help on "reform," we need not assert our opinions, but we can often quietly offer suggestions which are by no means fruitless. How many of our nurses have been called to care for those whose wish to pay a last tribute of respect to a dead friend has resulted in death to themselves; and yet few of us are brave enough to alter the old conventionality of keeping the head uncovered at the grave. And then, too, among the poor! Our district nurses know what "mourning" means to their slender resources—the rich must lead the way, and show that simple decency means respect—and a gruesome pageant mockery to our dead and the poor, to whom it all so often entails so much misery, will follow in their train.

